# Supplementary material for: Association between Mothers’ Emotional Problems and Autistic Children’s Behavioral Problems: The Moderating Effect of Parenting Style
Source: Int J Environ Res Public Health. 2023 Mar 5;20(5):4593. doi: 10.3390/ijerph20054593 (PMC10001708; doi:10.3390/ijerph20054593)
Supplement: Supplementary file 1 [file ijerph-20-04593-s001.zip › ijerph-2077511-supplementary.pdf]

Association between Mothers' Emotional Problems and Autistic Children's Behavioral Problems: The Moderating Effect of Parenting Style

Supplementary materials

Table S1 The prevalence of mother`s emotional problems and behavioral problems in children with autism

Variables		Total samples	%
SDQ_HA			
	Normal	14	17.5
	Borderline	13	16.3
	Abnormal	53	66.3
SDQ_ES			
	Normal	60	75
	Borderline	15	18.8
	Abnormal	5	6.3
SDQ_PB			
	Normal	12	15
	Borderline	6	7.5
	Abnormal	62	77.5
SDQ_PP			
	Normal	5	6.3
	Borderline	4	5
	Abnormal	71	88.8
SDQ_CP			
	Normal	50	62.5
	Borderline	17	21.3

	Abnormal	13	16.3
Maternal Depression			
	No	49	61.3
	Yes	31	38.8
Anxiety			
	No	50	62.5
	Yes	30	37.5

TableS2 Multivariate regression models of the predictors of SDQ scores

Model	SDQ-HA		SDQ-ES		SDQ-PB		SDQ-PP		SDQ-CP		SDQ-total scores	
	Adjusted β#	t	Adjusted β#	t	Adjusted β#	t	Adjusted β#	t	Adjusted β#	t	Adjusted β#	t
Model 1												
Supportive/engaged	-0.033	-0.247	-0.016	-0.115	0.158	1.298	0.093	0.669	-0.012	-0.092	0.114	0.877
Depression	**0.345**	**2.614***	-0.139	-1.01	-0.221	-1.839	0.161	1.171	0.305	2.284	0.176	1.367
Supportive/engaged* Depression	0.052	0.395	-0.122	-0.891	0.176	1.477	0.078	0.574	0.209	**1.579***	0.198	1.548
R2	0.087		0.083		0.242		0.013		0.066		0.199	
F	0.156		1.103		5.198		1.179		1.931		3.029	
Model 2												
Hostile/coercive	0.055	**0.47***	0.134	1.08	0.108	1.001	0.142	1.155	0.067	0.547	0.067	0.547
Depression	0.326	2.871	-0.126	-1.059	-0.339	-3.288	0.081	0.691	0.231	1.976	0.231	1.976
Hostile/coercive* Depression	0.113	0.987	-0.026	-0.221	-0.187	**-1.799***	-0.082	-0.694	0.109	0.923	0.109	0.923
R2	0.098		0.086		0.011		0.31		0.028		0.039	
F	2.438		1.145		1.145		5.468		1.376		1.53	
Model 3					-							
Supportive / engaged	-0.126	-1.117	0.069	0.575			0.022	0.18	-0.139	-1.173	0.05	0.446

Anxiety	0.343	**3.067****	-0.062	-0.523		0.078	0.661	0.162	1.388	0.105	0.961
Supportive / engaged* Anxiety	0.005	0.043	0.011	0.093		0.002	0.019	0.112	0.959	0.192	1.747
R2	0.108		-0.005			0.001		0.029		0.141	
F	2.592		0.931			1.012		1.389		3.165	
Model 4					-						
Hostile/coercive	0.083	0.704	0.118	0.95		0.149	1.208	0.09	0.728	0.263	**2.306***
Anxiety	0.345	**3.09****	-0.084	-0.715		0.058	0.501	0.155	1.316	0.054	0.502
Hostile/coercive* Anxiety	0.071	0.625	-0.024	-0.2		-0.04	-0.34	0.097	0.815	-0.099	-0.907
R2	0.105		0.002			0.02		0.008		0.167	
F	2.55		1.032			1.269		1.105		3.645	

Note: Adjusted variable: children age, children sex, family income

Table S3 Multivariate regression models of the predictors of SCQ scores

Model	SCQ-social		SCQ- communication		SCQ-replacatve		SCQ-total scores	
	Adjusted β#	t	Adjusted β#	t	Adjusted β#	t	Adjusted β#	t
Model 1								
Supportive/engaged	0.114	0.877	-0.273	-2.088	0.002	0.018	-0.212	-1.672
Depression	0.176	1.367	0.049	0.381	0.075	0.567	0.119	0.944
Supportive/engaged* Depression	0.198	1.548	0.05	0.391	0.107	0.813	0.132	1.059
R2	0.167		0.119		0.079		0.174	
F	1.045		2.782		2.137		3.778	
Model 2								
Hostile/coercive	0.149	1.208	0.004	0.033	0.175	1.482	-0.273	0.018
Depression	0.058	0.501	0.118	1.023	0.007	0.063	0.049	0.567
Hostile/coercive* Depression	-0.04	-0.34	0.198	1.704	0.009	0.074	0.05	0.813
R2	0.02		0.07		0.097		0.119	0.018
F	1.269		2.02		2.414		2.782	0.567
Model 3								
Supportive / engaged	-		-0.263	-2.396	-0.028	-0.247	-0.22	**-2.13***
Anxiety	-		0.204	1.884	0.135	1.195	0.285	**2.796****
Supportive / engaged* Anxiety	-		0.087	0.797	0.067	0.592	0.179	1.754
R2	-		0.161		0.091		0.259	
F	-		3.535		2.317		5.601	
Model 4								

Hostile/coercive	-	-0.005	-0.038	0.174	1.479	-0.038	-0.333
Anxiety	-	0.223	1.981	0.112	1.006	0.295	2.767**
Hostile/coercive* Anxiety	-	0.147	1.284	-0.04	-0.355	0.186	1.721
R2	-	0.09		0.111		0.185	

Note: Adjusted variable: children age, children sex, family income
